# Supplementary material for: Influence of Different Litter Regimens on Ceca Microbiota Profiles in Salmonella-Challenged Broiler Chicks
Source: Animals (Basel). 2025 Jul 11;15(14):2039. doi: 10.3390/ani15142039 (PMC12291792; doi:10.3390/ani15142039)
Supplement: Supplementary file 1 [file animals-15-02039-s001.zip › animals-3670368-supplementary.pdf]

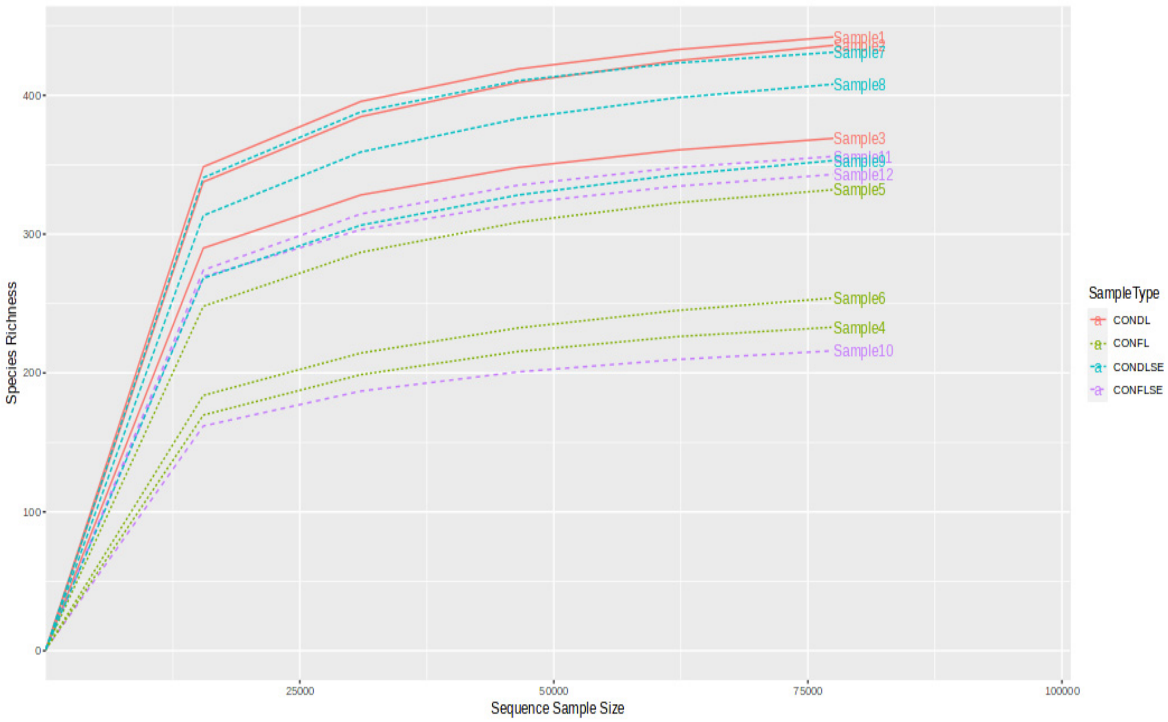

Figure S1 Rarefaction curves for all samples

Table S1 Phylum Relative Abundance

	SE Challenge			Litter Type		
**Phylum**	**NC**	**SE**	**P-Value**	**DL**	**FL**	**P-Value**
Actinobacteria	0.0011	0.0058	0.4140	0.0067	0.0002	0.2410
Bacteroidetes	0.0976	0.1879	0.1860	0.1357	0.1497	0.8450
Firmicutes	0.8347	0.7372	0.2600	0.7991	0.7729	0.7700
Lentisphaerae	0.0000	0.0010	0.3410	0.0010	0.0000	0.3410
Proteobacteria	0.0616	0.0530	0.8100	0.0466	0.0681	0.5420
**Tenericutes**	**0.0049**[b]	**0.0150**[a]	**0.0470**	0.0109	0.0091	0.7440

[a,b] Mean values bearing different superscript letters across the row are significantly different ($P < 0.05$). NC, no challenge, SE, Salmonella challenge, DL, Deep litter, Fl, fresh litter

Interaction Effect

Phylum	CONDL	CONFLSE	CONFL	CONDLSE	P-Value
Actinobacteria	0.0021	0.0002	0.0002	0.0114	0.4440
Bacteroidetes	0.0675	0.1718	0.1276	0.2040	0.5600
Firmicutes	0.8955	0.7718	0.7740	0.7026	0.4810
Lentisphaerae	0.0000	0.0000	0.0000	0.0020	0.4410
Proteobacteria	0.0279	0.0409	0.0954	0.0652	0.5560
Tenericutes	0.0070	0.0153	0.0028	0.0147	0.2730

CONDL, Unchallenged chicks on Dirty litter; CONDLSE, SE challenged chicks on Dirty litter; CONFL, Unchallenged chicks on Fresh litter; CONFLSE, SE challenged chicks on fresh litter

Table S2

Genera Relevance Abundance

	SE Challenge			Litter Type		
**Genera**	**noSE**	**SE**	**P-Value**	**DL**	**FL**	**P-Value**
Anaerofustis	0.0002	0.0005	0.3187	0.0003	0.0004	0.6560
Anaeroplasma	0.0013	0.0065	0.1229	0.0019	0.0059	0.2367
Anaerostipes	0.0001	0.0000	0.3893	0.0001	0.0000000	0.1945
Anaerotruncus	0.0006	0.0012	0.4605	0.0008	0.0011	0.7161
Bacteroides	0.0959	0.1862	0.1869	0.1340	0.1480	0.8445
Blautia	0.0475	0.0446	0.8324	0.0459	0.0461	0.9891
Brachybacterium	0.0002	0.0000	0.2870	0.0002	0.0000065	0.2765
Brevibacterium	0.0001	0.0000	0.1644	0.0001	0.0000022	0.1644
Campylobacter	0.0000	0.0003	0.3367	0.0000022	0.0003	0.3445
Candidatus_Arthromitus	0.0001	0.0004	0.1088	0.0001	0.0003	0.3706
**Cc_115**	**0.0070**[a]	**0.0012**[b]	**0.0239**	0.0042	0.0040	0.9363
Clostridium	0.0001	0.0001	0.5912	0.0002	0.0001	0.5145
Coprobacillus	0.0074	0.0069	0.8312	0.0063	0.0079	0.4910
Coprococcus	0.0132	0.0051	0.0661	0.0063	0.0120	0.2187
Corynebacterium	0.0003	0.0000	0.2873	0.0003	0.0000000	0.2007
Dehalobacterium	0.0000	0.0000	0.4158	0.0000345	0.0000000	0.2714
**Dorea**	**0.0112**[a]	**0.0072**[b]	**0.0390**	0.0079	0.0105	0.2131
Enterococcus	0.0144	0.0119	0.6694	0.0118	0.0145	0.6433
Eubacterium	0.0000	0.0002	0.3465	0.0002	0.0000022	0.3465
Faecalibacterium	0.0907	0.1244	0.5173	0.0813	0.1339	0.3044
Holdemania	0.0000	0.0000	0.3344	0.0000344	0.0000000	0.1891
Jeotgalicoccus	0.0000	0.0000	0.2769	0.0000215	0.0000000	0.1660
Lachnospira	0.0003	0.0003	0.9983	0.0006	0.0000344	0.0735
Lactobacillus	0.0778	0.0512	0.5191	**0.1156**[a]	**0.0133**[b]	**0.0020**
Nesterenkonia	0.0001	0.0000	0.3600	0.0001	0.0000000	0.3195
Not_Assigned	0.4071	0.3949	0.8094	**0.4469**[a]	**0.3552**[b]	**0.0433**
Oscillospira	0.0273	0.0298	0.7129	0.0262	0.0309	0.4903
Others	0.0000	0.0000	0.5118	0.0000409	0.0000129	0.3375
Peptococcus	0.0000	0.0018	0.3409	0.0018	0.0000000	0.3409
Proteus	0.0011	0.0006	0.5439	0.0006	0.0012	0.5107
Pseudoramibacter_Eubacterium	0.0001	0.0001	0.8275	0.0002	0.0001	0.2829
Roseburia	0.0001	0.0002	0.7877	**0.0003**[a]	**0.0000**[b]	**0.0208**
Ruminococcus	0.1955	0.1233	0.1622	**0.1056**[b]	**0.2132**[a]	**0.0244**
Slackia	0.0000	0.0001	0.3409	0.0001	0.0000000	0.3409
SMB53	0.0001	0.0010	0.3412	0.0001	0.0010	0.3184
Staphylococcus	0.0001	0.0001	0.9249	0.0001[a]	0.0000194[b]	**0.0191**
Vibrio	0.0000	0.0000	0.5954	0.0000172	0.0000043	0.4213
Yaniella	0.0001	0.0000	0.2952	0.0001	0.0000000	0.2952

[a,b] Mean values bearing different superscript letters across the row are significantly different (*P* < 0.05).
NC, no challenge, SE, Salmonella challenge, DL, Deep litter, Fl, fresh litter

Interaction Effect

Genera	CONDL	CONDLSE	CONFL	CONFLSE	P-Value
Anaerofustis	0.0003	0.0004	0.0002	0.0006	0.6723
Anaeroplasma	0.0010	0.0027	0.0016	0.0102	0.1582
Anaerostipes	0.0002	0.0000344	0.0000000	0.0000000	0.3672
Anaerotruncus	0.0001	0.0014	0.0011	0.0010	0.7200
Bacteroides	0.0675	0.2005	0.1243	0.1718	0.5761
Blautia	0.0417	0.0502	0.0533	0.0390	0.8754
Brachybacterium	0.0004	0.0000043	0.0000000	0.0000129	0.2931
Brevibacterium	0.0001	0.0000043	0.0000043	0.0000000	0.0992
Campylobacter	0.0000000	0.0000043	0.0000000	0.0006	0.4431
Candidatus_Arthromitus	0.0000129	0.0003	0.0001	0.0005	0.3371
Cc_115	0.0075	0.0009	0.0065	0.0015	0.2001
Clostridium	0.0002	0.0001	0.0000430	0.0002	0.1081
Coprobacillus	0.0068	0.0058	0.0080	0.0079	0.9178
**Coprococcus**	**0.0072**^{b}	**0.0054**^{b}	**0.0192**^{a}	**0.0048**^{b}	**0.0407**
Corynebacterium	0.0005	0.0000430	0.0000000	0.0000000	0.2536
Dehalobacterium	0.0000086	0.0001	0.0000000	0.0000000	0.4794
Dorea	0.0107	0.0052	0.0117	0.0093	0.0729
Enterococcus	0.0136	0.0100	0.0152	0.0138	0.9428
Eubacterium	0.0000000	0.0004	0.0000043	0.0000000	0.4442
Faecalibacterium	0.0872	0.0753	0.0942	0.1735	0.5368
Holdemania	0.0001	0.0000086	0.0000000	0.0000000	0.2991
Jeotgalicoccus	0.0000387	0.0000043	0.0000000	0.0000000	0.2081
Lachnospira	0.0006508	0.0005977	0.0000086	0.0001	0.4118
**Lactobacillus**	**0.1406**^{a}	**0.0907**^{a}	**0.0150**^{b}	**0.0116**^{b}	**0.0160**
Nesterenkonia	0.0001	0.0000043	0.0000000	0.0000000	0.4517
Not_Assigned	0.4620	0.4317	0.3521	0.3582	0.2742
Oscillospira	0.0274	0.0250	0.0272	0.0346	0.7934
Others	0.0001	0.0000129	0.0000043	0.0000215	0.4006
Peptococcus	0.0000000	0.0035	0.0000000	0.0000000	0.4411
Proteus	0.0012	0.0000000	0.0011	0.0012	0.7359
Pseudoramibacter_Eubacterium	0.0001	0.0002	0.0001	0.0000172	0.6925
Roseburia	0.0002	0.0003	0.0000258	0.0000000	0.1561
**Ruminococcus**	**0.1222**^{b}	**0.0889**^{b}	**0.2688**^{a}	**0.1576**^{ab}	**0.0308**
Slackia	0.0000000	0.0002	0.0000000	0.0000000	0.4411
SMB53	0.0001	0.0000043	0.0000473	0.0020	0.3767
Staphylococcus	0.0001	0.0001	0.0000215	0.0000172	0.1808
Vibrio	0.0000301	0.0000043	0.0000000	0.0000086	0.5696
Yaniella	0.0001	0.0000000	0.0000000	0.0000000	0.3421

^{a,b} Mean values bearing different superscript letters across the row are significantly different ($P < 0.05$).
CONDL, Unchallenged chicks on Dirty litter; CONDLSE, SE challenged chicks on Dirty litter; CONFL, Unchallenged chicks on Fresh litter; CONFLSE, SE challenged chicks on fresh litter

Table S3 TopTen Genus

Salmonella Enteritidis Challenge:

	**SE Challenge**			**Litter Type**		
**Genera**	NC	SE	P-Value	**DL**	**FL**	**P-Value**
Bacteroides	0.0959	0.1862	0.1869	0.1340	0.1480	0.8445
Blautia	0.0475	0.0446	0.8324	0.0459	0.0461	0.9891
Coprococcus	0.0132	0.0051	0.0661	0.0063	0.0120	0.2187
**Dorea**	**0.0112**^{a}	**0.0072**^{b}	**0.0390**	0.0079	0.0105	0.2131
Enterococcus	0.0144	0.0119	0.6694	0.0118	0.0145	0.6433
Faecalibacterium	0.0907	0.1244	0.5173	0.0813	0.1339	0.3044
Lactobacillus	0.0778	0.0512	0.5191	**0.1156**^{a}	**0.0133**^{b}	**0.0020**
Not_Assigned	0.4071	0.3949	0.8094	**0.4469**^{a}	**0.3552**^{b}	**0.0433**
Oscillospira	0.0273	0.0298	0.7129	0.0262	0.0309	0.4903
Others	0.0195	0.0214	0.7383	0.0185	0.0224	0.5001
Ruminococcus	0.1955	0.1233	0.1622	**0.1056**^{b}	**0.2132**^{a}	**0.0244**

^{a,b} Mean values bearing different superscript letters across the row are significantly different ($P < 0.05$).
NC, no challenge, SE, Salmonella challenge, DL, Deep litter, Fl, fresh litter

Interaction Effect

**Genera**	**CONDL**	**CONDLSE**	**CONFL**	**CONFLSE**	**P-Value**
Bacteroides	0.0675	0.2005	0.1243	0.1718	0.5761
Blautia	0.0417	0.0502	0.0533	0.0390	0.8754
**Coprococcus**	**0.0072**^{b}	**0.0054**^{b}	**0.0192**^{a}	**0.0048**^{b}	**0.0407**
Dorea	0.0107	0.0052	0.0117	0.0093	0.0729
Enterococcus	0.0136	0.0100	0.0152	0.0138	0.9428
Faecalibacterium	0.0872	0.0753	0.0942	0.1735	0.5368
**Lactobacillus**	**0.1406**^{a}	**0.0907**^{a}	**0.0150**^{b}	**0.0116**^{b}	**0.0160**
Not_Assigned	0.4620	0.4317	0.3521	0.3582	0.2742
Oscillospira	0.0274	0.0250	0.0272	0.0346	0.7934
Others	0.0201	0.0169	0.0189	0.0259	0.7394
**Ruminococcus**	**0.1222**^{b}	**0.0889**^{b}	**0.2688**^{a}	**0.1576**^{ab}	**0.0308**

^{a,b} Mean values bearing different superscript letters across the row are significantly different ($P < 0.05$).
CONDL, Unchallenged chicks on Dirty litter; CONDLSE, SE challenged chicks on Dirty litter; CONFL, Unchallenged chicks on Fresh litter; CONFLSE, SE challenged chicks on fresh litter
